# Local ubiquitin-proteasome-mediated proteolysis and long-term synaptic plasticity

**DOI:** 10.3389/fnmol.2014.00096

**Published:** 2014-12-01

**Authors:** Ashok N. Hegde, Kathryn A. Haynes, Svitlana V. Bach, Brenna C. Beckelman

**Affiliations:** Department of Neurobiology and Anatomy, Wake Forest University Health SciencesWinston-Salem, NC, USA

**Keywords:** ubiquitin, proteasome, learning and memory, protein degradation, ubiquitin conjugation

## Abstract

The ubiquitin-proteasome pathway (UPP) of protein degradation has many roles in synaptic plasticity that underlies memory. Work on both invertebrate and vertebrate model systems has shown that the UPP regulates numerous substrates critical for synaptic plasticity. Initial research took a global view of ubiquitin-protein degradation in neurons. Subsequently, the idea of local protein degradation was proposed a decade ago. In this review, we focus on the functions of the UPP in long-term synaptic plasticity and discuss the accumulated evidence in support of the idea that the components of the UPP often have disparate local roles in different neuronal compartments rather than a single cell-wide function.

Many years of research on synaptic plasticity using various model systems, from slugs and flies to mammals, has yielded a wealth of information on mechanisms that underlie both short- and long-term synaptic plasticity (Kandel and Schwartz, 1982; Belvin and Yin, 1997; Mayford, 2007; Walters and Moroz, 2009; Abrams, 2012). It is now generally accepted that short-term synaptic plasticity requires modification, usually by phosphorylation, of pre-existing proteins. Long-term plasticity, however, requires gene transcription and translation of newly transcribed mRNAs (Hernandez and Abel, 2008; Sossin, 2008; Katche et al., 2013). Research over the last couple of decades has revealed another major mechanism with roles in both short- and long-term synaptic plasticity: protein degradation by the ubiquitin-proteasome pathway (UPP; Hegde, 2010; Fioravante and Byrne, 2011; Jarome and Helmstetter, 2013).

Previously one of us proposed a role for local proteolysis by the UPP in synaptic plasticity (Hegde, 2004). The gist of this theory is that proteolysis by the UPP performs disparate functions in different parts of the neuron. Since then, investigations on different model systems have obtained evidence to support the function of local proteolysis. The idea has gained acceptance by other researchers as well (Segref and Hoppe, 2009). In the nervous system, to achieve synapse-specific effects, proteolysis needs to be spatially restricted. Therefore, local protein degradation is likely to be critical during development of synaptic connections as well as synaptic plasticity in adult organisms.

How might local protein degradation be achieved in neurons? A simple way would be to restrict the protein substrate or the enzymes of the UPP to a subcellular location. For example, proteins whose expression is largely restricted to the synapses could be locally degraded because all the requisite UPP components are present at the synapse. In addition, substrates can be made susceptible (or resistant) to ubiquitination by locally controlled phosphorylation in neurons. Likewise ubiquitin ligases can be activated or inactivated locally by phosphorylation or other posttranslational modifications (e.g., conjugation of ubiquitin-like protein Nedd8 to Cul1 that activates SCF ligases; Osaka et al., 2000; Lyapina et al., 2001) can be locally controlled as well (Hegde and Upadhya, 2007; Hegde, 2010). Moreover, specific E3 ligases can also be sequestered to specific cellular compartments (Tsai et al., 2012; Ichimura et al., 2013). Experimental evidence has been obtained for some of these possibilities. Evidence gathered over recent years indicates that proteasome activity is differentially regulated in different neuronal compartments as well. A few examples of local roles of ubiquitination and those of the proteasome in neuronal compartments are discussed below.

## ROLES FOR LOCAL UBIQUITINATION AND DEUBIQUITINATION

As described previously, the specificity of ubiquitination is largely controlled at the level of E3 ubiquitin ligases (Glickman and Ciechanover, 2002). The specificity of ubiquitination could also be regulated at the level of E2s because E2s are diverse and many unique E2–E3 combinations can be generated (Glickman and Ciechanover, 2002; Hegde, 2010). Several studies show evidence for local roles of E2s and E3s as well as for deubiquitinating enzymes (DUBs) during development of synaptic connections.

### E2s

In *Drosophila*, an E2 called ubcD1 controls dendritic pruning where local degradation appears to be critical. In this insect, most of the larval neurons die during metamorphosis, except for a cluster of peripheral sensory neurons named class IV dendritic arborization (C4da) neurons which survive to adulthood (Kuo et al., 2005). These neurons extensively remodel their dendrites by completely degrading the old arborization and by growing a new elaborate set of dendrites. During the remodeling of dendrites, axons are kept intact. Hence the molecular processes have to be spatially restricted. It was found that perturbations of the UPP by overexpression of an exogenous DUB called UBP2 from yeast, or mutations in E1 or a 19S proteasome subunit disrupted dendritic pruning. Subsequent studies identified the essential role of ubcD1 in this process (Kuo et al., 2006). Mutations in ubcD1 led to a blockade of dendritic pruning and retention of larval dendrites in C4da neurons. Based on additional experiments it was inferred that ubcD1 targets *Drosophila* inhibitor of apoptosis 1 (DIAP1) an E3 ubiquitin ligase. DIAP1 is required for degradation of a caspase called Dronc. Therefore, degradation of DIAP1 enables activation of the Dronc caspase locally in dendrites. Because the Dronc caspase is critical for severing dendrites of C4da neurons, restricted dendritic activation of this caspase allows preservation of C4da neurons while removing their dendrites (Kuo et al., 2005, 2006).

### E3s

A genetic screen for isolating mutants that enhance synaptic growth at the *Drosophila* neuromuscular junction revealed that a loss-of-function mutation in a gene called *highwire* (*hiw*) causes a substantial increase in number of synapses. The protein product of the *hiw* gene contains a RING finger domain which is a key part of some ubiquitin ligases (Wan et al., 2000).

Subsequent work carried out on the *Caenorhabditis elegans* homolog of the *hiw* gene called RPM-1 showed that the ligase functions to regulate presynaptic differentiation. RPM-1 protein is localized to the periactive zone, a presynaptic region excluded from the active zone and synaptic vesicles. RPM-1 combines with an F-box protein called FSN-1 and *C. elegans* homologs of SKP1 and Cullin to form an SCF-like ubiquitin ligase complex. The localized function of this ubiquitin ligase in the periactive zone is critical for presynaptic differentiation in *C. elegans* (Liao et al., 2004). The downstream target of RPM-1 in *C. elegans* is a MAP kinase kinase kinase (MAPKKK) called delta-like homolog 1 (DLK-1) which is also localized to the periactive zone like RPM-1. Inactivation of the DLK-1 cascade suppresses RPM-1 loss-of- function phenotypes whereas DLK-1 overexpression causes synaptic aberrations similar to the ones seen with RPM-1 mutations (Nakata et al., 2005). In *Drosophila*, the downstream target of hiw is a MAPKKK encoded by a gene called *wallenda* (Collins et al., 2006). Although the downstream effectors of DLK-1 and wallenda proteins are different, attenuation of the signaling mediated by these proteins inhibits synaptic growth in similar ways (Fulga and Van, 2008).

In vertebrates, a homolog of *hiw* called *Phr1* regulates development of neuronal connections. Studies using mice with a mutation in the *Phr1* gene (a mutation called *Magellan*), which lacks the *C*-terminal ligase domain, revealed that the Phr1 protein is localized to axon shaft and is largely excluded from growth cones and distal processes. The substrate of Phr1 is most likely DLK in mice as well. Distribution of DLK is non-overlapping with that of Phr1; DLK is present in growth cones with only low levels in axon shaft (Lewcock et al., 2007).

Local regulation of other E3 ligases has also been reported. For example, in hermaphrodite-specific motor neurons of *C. elegans*, an SCF ligase containing the protein SKR-1 and an F-box protein called SEL-10 mediates developmental elimination of synapses. A synaptic adhesion molecule called SYG-1 binds to SKR-1 and blocks the assemblage of the SCF complex which protects the nearby synapses (Ding et al., 2007). In vertebrates, the ligase anaphase promoting complex containing the substrate-binding protein Cdh1 curtails axonal growth when it is nuclear, but when it is cytosolic it promotes dendrite growth without affecting axons (Kim et al., 2009). Another ligase KLHL20-Cullin 3 promotes neurotrophin-induced neurite outgrowth by targeting RhoGEF for proteolysis (Lin et al., 2011).

### DEUBIQUITINATING ENZYMES

Ubiquitination can be reversed by removal of the attached ubiquitin molecules by DUBs. Thus DUBs provide important negative regulation of protein degradation. Like ligases, DUBs can act locally to reverse ubiquitination. A search for molecules that regulate the size and strength of synapses in *Drosophila* found that a DUB encoded by the *fat facets (faf)* gene functions in synapse formation. During development of the *Drosophila* nervous system, *faf* overexpression leads to overgrowth of synapses and disruption of synaptic transmission. A similar phenotype is observed when a yeast DUB is expressed in the fruit fly CNS (DiAntonio et al., 2001).

Deubiquitinating enzymes also have a local synaptic role in mammals. A DUB called Usp14 is essential for synaptic development and function in mouse neuromuscular junctions. The role for Usp14 in the nervous system was originally discovered through studies on mice with the *ataxia* (*ax^j^*) mutation, a recessive mutation characterized by severe tremors, hind limb paralysis, and postnatal lethality (Wilson et al., 2002). The *ax^j^* gene encodes Usp14 the protein product of which associates with the proteasome and is believed to help disassemble polyubiquitin chains and recycle ubiquitin thus maintaining ubiquitin levels in the cell. Accordingly, loss of Usp14 results in reduced ubiquitin levels in many tissues of the *ax^j^* mice including the brain (Anderson et al., 2005). The motor defects of the *ax^j^* mice were rescued and viability was restored with transgenic Usp14 suggesting that Usp14 deficiency is the cause of neurological defects in these mice (Crimmins et al., 2006). Subsequent studies demonstrated that in Usp14-deficient *ax^j^* mice ubiquitin loss occurred in the spinal cord and sciatic nerve. Biochemically, the majority of the loss was found to occur in synaptosomal fractions indicating that Usp14 at synaptic sites was critical. Loss of Usp14 caused presynaptic defects such as poor arborization of motor nerve terminals, and transgenic expression of Usp14 rescued these defects. Thus it appears that local Usp14 function is critical for maintaining ubiquitin levels and hence protein degradation at the synapse (Chen et al., 2009).

## LOCAL ROLES OF THE PROTEASOME IN SYNAPTIC PLASTICITY

Recently it has become clear that the proteasome is not the same in terms of its activity and function throughout the neuron. This realization came from attempts to resolve conflicting results obtained with proteasome inhibitors on long-term facilitation (LTF) in *Aplysia*. Initially, proteasome inhibitors were found to block induction of LTF (Chain et al., 1999). Later studies on LTF, however, showed that when the active form of lactacystin, *clasto*-lactacystin β-lactone (hence forth referred to as β-lactone), was applied in the culture medium to sensory-motor neuron synapses, this resulted in enhanced LTF and an increase in neurite outgrowth in isolated sensory neuron (Zhao et al., 2003). The results on enhanced neurite elongation are consistent with those obtained in PC12 and Neuro2A cells in which lactacystin induces neurite outgrowth (Fenteany et al., 1994). The discrepancies between these results can be resolved by hypothesizing that the proteasome has different roles in different cellular compartments (Hegde, 2004). In a given neuron, the proteasome is likely to carry out dissimilar tasks in various subcellular compartments resulting in distinct physiological outcomes at separate neuronal locales. Thus, blocking discrete roles of the proteasome during induction of memory would lead to distinctive and even opposite effects on synaptic strength. For example, the proteasome degrades transcription repressors. Proteolytic removal of transcription repressors should enable transcription activators to induce gene expression and consequent development of LTF. If the proteasome is inhibited only in the nucleus before the repressors are degraded, gene expression and hence induction of LTF should be blocked. Degradation of the CREB repressor CREB1b by the UPP in response to LTF-inducing protocols (Upadhya et al., 2004) supports this idea. In contrast, if the proteasome is inhibited at the synapse causing accumulation of LTF-inducing proteins, LTF should be augmented. As postulated earlier, transcription is necessary during induction of LTF (or other forms of plasticity underlying long-term memory) for supplying mRNAs for synthesis of proteins that turn over rapidly in the early stages of memory formation (Hegde, 2004). Blocking the degradation of such proteins should cause long-term memory to form without transcription. Consistent with this notion, it was found that proteasome inhibitor-induced synaptic strengthening depends on translation but not transcription (Zhao et al., 2003).

Results from direct measurement of proteasome activity also support differential function of the proteasome in distinct neuronal compartments. Proteasome activity in the synaptic terminals is much higher compared to that in the nucleus in *Aplysia* nervous system as well as the mouse brain. Furthermore, the synaptic and nuclear proteasome activities are differentially regulated by protein kinases with a key role in synaptic plasticity such as PKA, PKC, and MAP kinase (Upadhya et al., 2006). Recently others have found that CaMKII can stimulate proteasome activity in cultured hippocampal neurons (Djakovic et al., 2009).

As discussed above, differential proteasome activity in the invertebrate *Aplysia* might explain conflicting results obtained in different studies. Does the idea of proteasomal activity affecting synaptic plasticity differentially hold true for vertebrates? It has been observed that the proteasome has differential roles during the induction and maintenance parts of late-phase long-term potentiation (L-LTP) in the murine hippocampus (Dong et al., 2008), which is discussed in detail in the next section.

The proteasome has been shown to be dynamically and locally regulated at the dendrites in cultured rat hippocampal neurons. In response to NMDA receptor activation proteasome was found to be redistributed from dendritic shafts to synaptic spines. What is the mechanism of redistribution of the proteasome? Neuronal activity increased the entry of the proteasome into dendritic shafts only to a small extent but drastically reduced their exit. In addition, proteasome was found to be sequestered persistently in the spines through association with cytoskeleton (Bingol and Schuman, 2006). Later investigations showed that a protein called NAC1, which is induced by psychostimulants, modulates the recruitment of the proteasome into the dendritic spines (Shen et al., 2007). Much of the evidence from these studies, however, was on the catalytic 20S core of the proteasome. Thus, it is not clear whether recruitment of the full 26S proteasome complex that degrades polyubiquitinated proteins is also regulated by NAC1. A recent study has suggested that the CaMKIIα subunit acts as a scaffold for the proteasome (Bingol et al., 2010). It is not clear how or if the functions of NAC1 and CaMKIIα relate to each other in sequestering the proteasome. Local proteolysis by the proteasome also has been shown to be critical for regulating spine outgrowth (Hamilton et al., 2012).

It is also likely that the proteasome operates locally to regulate other molecular processes required for synaptic plasticity such as translation of mRNA. For instance, proteasome is known to regulate fragile X mental retardation protein (FMRP), which modulates translation of a subset of mRNAs in dendrites. Moreover, proteasomal regulation of FMRP is required for metabotropic glutamate receptor-dependent LTD (Hou et al., 2006).

### DISPARATE LOCAL ROLES OF THE PROTEASOME IN DENDRITES AND THE NUCLEUS: OPPOSITE CONSEQUENCES FOR INDUCTION AND MAINTENANCE OF L-LTP

**FIGURE 1:**
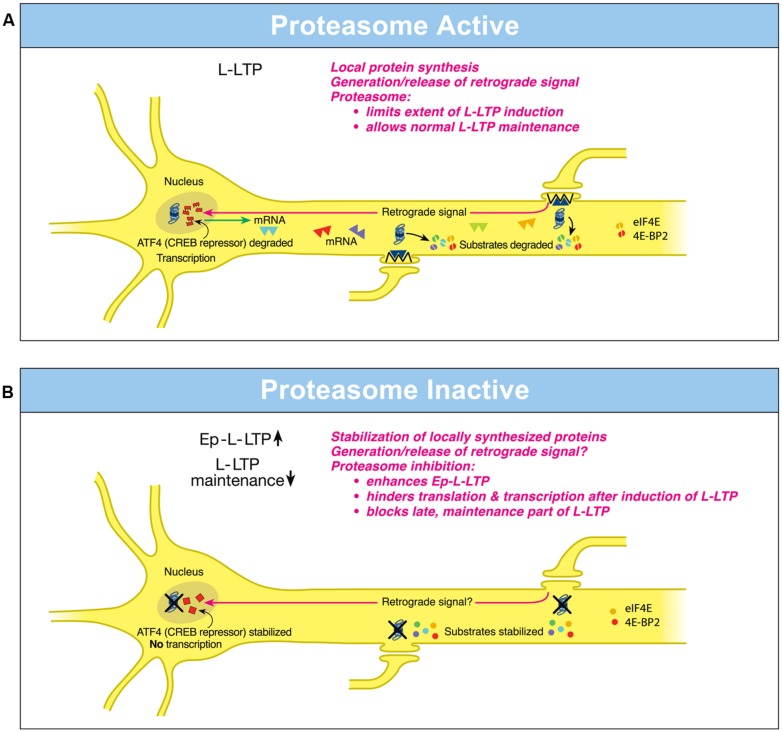
**Differential local roles of the proteasome in dendrites and in the nucleus during L-LTP. (A)** Proteasome Active: the proteasome in dendrites is highly active, translational activators such as eIF4E are degraded (broken green spheres) and protein substrates that positively regulate L-LTP are degraded (broken spheres). Therefore extent of L-LTP is limited and only normal L-LTP ensues. A retrograde signal is likely transmitted to the nucleus. Proteasome aids transcription of genes by degrading the CREB repressor ATF4 (broken squares in the nucleus) thus allowing for normal L-LTP maintenance. Transcribed mRNAs (triangles) travel to activated synapses. **(B)** Proteasome Inactive: when the proteasome is inhibited (indicated by X marks on the proteasome), translational activators are stabilized (intact green spheres) leading to increased protein synthesis in dendrites. Also the newly synthesized proteins in dendrites are stabilized (intact spheres) and L-LTP-inducing stimulation protocols dramatically increase (upward arrow) the early part of L-LTP (Ep-L-LTP). Proteasome inhibition obstructs CREB-mediated transcription by preventing the degradation of transcription repressor ATF4 (intact squares in the nucleus). Proteasome inhibition could also inhibit the generation of the retrograde signal. Therefore, L-LTP is not maintained but decays (downward arrow). Proteasome inhibition also causes failure of sustained translation because of stabilization of translation repressors such as 4E-BP (intact red spheres) which accumulate after induction of L-LTP thus contributing to blockade of L-LTP maintenance. [Modified from Hegde (2010) and reprinted with permission from Cold Spring Harbor Laboratory Press].

Investigations on hippocampal L-LTP showed that the proteasome inhibitor application to hippocampal slices prior to induction of L-LTP caused an increase in the magnitude of the early, induction phase but an inhibition of the late, maintenance phase (Dong et al., 2008). What is the basis of these differential effects of the proteasome on phases of L-LTP? The enhancement of the early part of L-LTP (referred to as Ep-L-LTP for convenience) by the proteasome inhibitor β-lactone is blocked by prior application of the translation inhibitor anisomycin but not by a transcription inhibitor actinomycin D. The increase in Ep-L-LTP caused by β-lactone is also prevented by prior application of rapamycin which blocks signaling that controls translation of a subset of mRNAs (Gingras et al., 2001). Moreover, Ep-L-LTP is augmented by β-lactone in dendrites isolated from the cell body by means of a surgical cut. These lines of evidence suggest that proteasome inhibition enhances Ep-L-LTP by stabilizing proteins locally translated from pre-existing mRNAs (Dong et al., 2008; **Figure 1A**).

How does proteasome inhibition block maintenance of L-LTP? The proteasome inhibitor β-lactone blocks maintenance of L-LTP only if applied prior to induction of L-LTP but not if applied 2 h after induction of L-LTP. Previous studies by others have established that the critical time window for transcription required for maintenance of L-LTP is 2 h (Nguyen et al., 1994). These results suggest that proteasome inhibition blocks maintenance of L-LTP by inhibiting transcription. Additional molecular evidence supports this notion. Application of β-lactone to hippocampal slices significantly reduced induction of *brain-derived neurotrophic factor* (*BDNF*) mRNA by chemically induced LTP (cLTP) or L-LTP induced by a theta-burst protocol (Dong et al., 2008). *BDNF* is a CREB-inducible gene linked to maintenance of L-LTP (Barco et al., 2005).

How might proteasome inhibition block transcription? One possibility is that normally the UPP aids the degradation of transcription repressors. Hence proteasome inhibition would result in accumulation of these repressors thus blocking transcription. Consistent with this concept, it was found that a CREB repressor ATF4 is degraded by the UPP during cLTP and β-lactone application to hippocampal slices prevents degradation of ATF4. Furthermore, ATF4-ubiquitin conjugates accumulate during cLTP when the proteasome is inhibited (Dong et al., 2008; **Figure 1B**).

These studies have also revealed the changing role of the proteasome even in dendrites through progression of L-LTP. Application of β-lactone to isolated dendrites also blocks maintenance of the dendritic L-LTP (Dong et al., 2008). Under these conditions, there is no supply of newly transcribed mRNA from the cell body. Thus blockade of transcription by proteasome inhibition does not explain this phenomenon. The most likely possibility is that proteasome inhibition leads to a slow accumulation of translation repressors in dendrites. Buildup of translation repressors would also occur in the cell body which would hinder translation of newly transcribed mRNAs. Thus late stages of translation in both dendrites and the cell body would be blocked by stabilization of translation repressors by proteasome inhibition. In support of this idea, confocal microscopy experiments at various time points after L-LTP induction showed that proteasome inhibition causes accumulation of translational activators eukaryotic initiation factors 4E (eIF4E) and eukaryotic elongation factor 1A (eEF1A) early during L-LTP (Dong et al., 2014). Translational repressors such as polyadenylate-binding protein interacting protein 2 (Paip2) and eukaryotic initiation factor 4E-binding protein 2 (4E-BP2) buildup at later stages of L-LTP in response to proteasome inhibition (Dong et al., 2014). Other negative regulators of translational repressors such as Mov10 might be stabilized by proteasome inhibition as well. For example, in cultured hippocampal neurons Mov10, which inhibits translation of key plasticity-related mRNAs such as that of *CaMKIIα,* is degraded by the proteasome in an NMDA- and activity-dependent manner (Banerjee et al., 2009).

Other studies have investigated the effect of proteasome inhibition on LTP. These studies failed to discern differential roles of the proteasome in LTP because one investigation used MG-132 (Karpova et al., 2006) which is not a highly specific proteasome inhibitor (Chain et al., 1999; Tang and Leppla, 1999) and the other utilized proteasome inhibitors lactacystin and epoxomicin at nanomolar concentration (Fonseca et al., 2006) which is substantially lower than the effective concentration (micromolar) essential to block proteasome activity.

## FUTURE DIRECTIONS

The evidence accumulated over the past decade has further supported the importance of local protein degradation in synaptic plasticity during brain development as well as in the adult brain. What has been lacking is research on the possible mechanisms by which local proteolysis is regulated in neurons. Looking ahead, we can expect to see exciting new discoveries on the local roles of protein degradation in the normal nervous system as well as in many neurodegenerative diseases.

## Conflict of Interest Statement

The authors declare that the research was conducted in the absence of any commercial or financial relationships that could be construed as a potential conflict of interest.
